# New Insights Into the Role of Autoreactive CD8 T Cells and Cytokines in Human Type 1 Diabetes

**DOI:** 10.3389/fendo.2020.606434

**Published:** 2021-01-05

**Authors:** Christine Bender, Sakthi Rajendran, Matthias G. von Herrath

**Affiliations:** Center for Autoimmunity and Inflammation, Type 1 Diabetes Center at La Jolla Institute for Immunology, La Jolla, CA, United States

**Keywords:** type 1 diabetes, autoreactive CD8 T cells, human pancreas, beta cells, cytokines

## Abstract

Since the establishment of the network for pancreatic organ donors with diabetes (nPOD), we have gained unprecedented insight into the pathology of human type 1 diabetes. Many of the pre-existing “dogmas”, mostly derived from studies of animal models and sometimes limited human samples, have to be revised now. For example, we have learned that autoreactive CD8 T cells are present even in healthy individuals within the exocrine pancreas. Furthermore, their “attraction” to islets probably relies on beta-cell intrinsic events, such as the over-expression of MHC class I and resulting presentation of autoantigens such as (prepro)insulin. In addition, we are discovering other signs of beta-cell dysfunction, possibly at least in part due to stress, such as the over-expression of certain cytokines. This review summarizes the latest developments focusing on cytokines and autoreactive CD8 T cells in human type 1 diabetes pathogenesis.

## Introduction

Type 1 diabetes (T1D) is a T cell-mediated autoimmune disease in which the pancreatic insulin-secreting beta cells are selectively destroyed. From decades of T1D studies performed in mice, profound insulitis was thought to be a common feature of T1D. However, with more extensive recent histological studies in the human pancreata, it is evident that insulitis is not that pronounced in patients with T1D (1). Autoreactive CD8 and CD4 T cells are critical players in beta-cell destruction (2) as they recognize peptides of beta-cell antigens that include (pro)insulin and its precursor preproinsulin (PPI), tyrosine phosphatase-like insulinoma antigen (IA-2), islet-specific glucose-6-phosphate catalytic subunit-related protein (IGRP), glutamic acid decarboxylase-65 (GAD65), zinc transporter protein 8 (ZnT8), and islet amyloid polypeptide (IAPP) (3–9). While autoreactive CD8 T cells are activated through interaction with peptides presented by HLA class I (e.g., HLA-A*02:01, -A*24:02, and -B*39:06) (3, 6, 10), CD4 T cells recognize peptides presented by HLA class II molecules (e.g., HLA-DR4, -DQ8) (8, 11, 12). However, it remains unexplored how they cooperate in islet destruction during T1D development as visualizing T cell responses in the human pancreas *in vivo* remains challenging. Nevertheless, mouse studies suggest that CD4 T cells provide help to effector CD8 T cells, stimulate antibody production by B cells, and activate islet-resident macrophages (13, 14). In addition, intravital two-photon microscopy in a mouse model of autoimmune diabetes indicates a crucial role of CD4 T helper cells in sustaining effector functions of cytotoxic CD8 T cells at target sites (13).

It is not clearly known what microenvironmental cues drive autoreactive T cells near the islets in the context of T1D. It is thought that factors such as cytokines, chemokines, oxidative stress, and altered antigen presentation may stimulate CD8 T cells, which were previously ignoring self-antigens, to activate and expand. The fact that the autoreactive T cells are also present in the pancreata of healthy donors (15) suggests a possibility of a triggering event in beta cells, which leads to the recruitment of these cells to the islets in T1D. In this review, we will discuss beta cell-intrinsic events that may increase the visibility of beta cells to the immune system and contribute to the recruitment of autoreactive T cells to the islets.

## Mini-Review

### What Distinguishes T1D Patients From Healthy Individuals?

For several years, researchers studied T cell responses against islet and beta-cell antigens in peripheral blood to find signatures that differentiate between T1D patients and healthy individuals. Initial studies suggested that autoreactive T cells escape negative selection (16, 17) in the thymus and are released into the peripheral blood in patients with T1D, but not in healthy individuals. Then, they get activated by antigen-presenting cells in the draining lymph nodes, which present epitopes derived from islet antigens. This hypothesis was supported by the linkage of polymorphism in the *INS* gene locus that determines the level of insulin expression in the thymus (17). However, many studies showed that autoreactive T cells are present in healthy individuals’ peripheral blood (4, 6), refuting the belief that autoreactive T cells escape negative selection only in T1D patients. Thus, it raises the question of whether the frequencies of such autoreactive T cells in peripheral blood can help identify patients with T1D. Indeed, higher frequencies of circulating beta-cell specific CD8 T cells were detected in HLA-A*02:01^+^ or HLA-A*24:02^+^ subjects with T1D compared with healthy donors (3, 7). Among them, PPI-reactive CD8 T cells were more frequently found in T1D patients than in healthy donors (4, 5, 7). On the other hand, other studies demonstrated no significant differences between T1D and control individuals in the frequencies of circulating CD8 T cells reactive to multiple A*02:01-restricted beta-cell epitopes (PPI_14-25_, PPI_6-14_, InsB_10-18_, GAD_114-123_, IA-2_797-805_, IA-2_805-813_, IGRP_265-273_, ZnT8_186-194_) (3, 4, 6, 10). However, beta cell-specific CD8 T cells are more differentiated in patients with newly diagnosed T1D compared to healthy controls (6), and memory T cell subsets were enriched within PPI_5-12_-specific CD8 T cell populations in HLA-B*39:06^+^ children with newly diagnosed T1D, but not in healthy control subjects (10). Overall, these studies suggest that phenotypic subset analysis of such circulating autoreactive T cells could help to identify patients with T1D.

### The Presence of Autoreactive CD8 T Cells in the Target Organ

The primary critique of studies performed using peripheral blood is that the relationship between tissue-infiltrating and circulating autoreactive CD8 T cells is poorly defined. Many studies of postmortem samples of pancreata revealed the dominant presence of CD8 T cells in donors with T1D and healthy pancreas (18–20). Using HLA class-I tetramers, Coppieters et al. provided the first proof that CD8 T cells reactive against IGRP_265-273_, IA-2_797-805_, and PPI_15-24_ could be found *in situ* in the islets from individuals with recent-onset and long-standing T1D (21). In these cases, it appeared that early after diagnosis, the islets contained CD8 T cells specific for one particular autoantigen, whereas, in patients with long-standing T1D, those islets with insulitis showed T cells with multiple specificities. Building on these findings, Bender et al. further examined the precise localization of PPI_15-24_-reactive CD8 T cells not only in the pancreas of nPOD donors with T1D but also in autoantibody-positive and healthy controls (15). Notably, the study showed that many PPI_15-24_-specific CD8 T cells are present in the exocrine pancreas of healthy donors and donors with autoantibodies supporting the theory of a general leakiness of central tolerance. In T1D patients, these cells were not only enriched in the exocrine pancreas but were also present within the islets or close to the islets (15). The PPI-specific CD8 T cells significantly infiltrated insulin-containing islets suggesting a critical effector mechanism leading to beta-cell destruction. In line with this, *in-situ* staining of nPOD pancreas sections revealed similar numbers of ZnT8_186-194_-positive A*02:01-restricted cells in the pancreas of healthy and autoantibody-positive donors but enriched in the pancreas of donors with T1D (4). Surprisingly, the frequencies of PPI_15-24_-reactive CD8 T cells detected in the exocrine pancreas were similar irrespective of disease status. Remarkably, many CD8 T cells recognized the PPI _15-24_ epitopes (15). In contrast to previous studies, where less than 1% of autoreactive CD8 T cells were detected within the peripheral blood (4, 5), frequencies in the pancreas are much higher (30-40%) (15). Besides, the majority of PPI-reactive CD8 T cells in the exocrine pancreas were positive for CD45RO, suggesting an antigen-experienced phenotype.

Besides conventional peptides, CD8 T cells can also recognize post-translationally modified peptides, IAPP_15-17_/IAPP_5-10,_ and SCG-009_186–194_ generated by mRNA splicing and transpeptidation, respectively (9). Gonzalez-Duque et al. revealed an enriched presence of IAPP_15-17_/IAPP_5-10_, urocortin 3 (UCN3)_1-9_, and transcription factor ISL1_276-284_ reactive cells in the human pancreas of donors with T1D (9). In comparison, the frequency of these reactive cells was similar between T1D patients and healthy individuals (9).

Although CD8 T cells are the most predominant immune infiltrates in human insulitis [1], autoreactive CD4 T cells are also involved in the pathogenesis of T1D and CD4 T cell responses to proinsulin, GAD, and IA-2 were identified in the islets of donors with T1D (8, 22, 23).

Consequently, what determines the progression of T1D in the face of similar frequencies of autoreactive CD8 T cells? Do beta-cells respond differently to inflammation that causes the attraction of CD8 T cells? The high numbers of autoreactive CD8 T cells in the pancreas suggest that autoreactivity is physiological and that disease development, therefore, is prevented under normal circumstances by local, organ-specific control mechanisms. Thus, defective organ-specific control mechanisms and/or a pro-inflammatory islet microenvironment are key pathogenic features of T1D.

### How Stressed Beta Cells Secrete Cytokines That May be Involved in Bringing in Autoreactive T Cells?

Cytokine and chemokine secretion are broadly implicated as key immune cell recruiting factors in T1D. It is believed that cytokines are typically secreted by immune cells during the pathogenesis of T1D. However, recently mounting evidence suggests that pancreatic islets per se secrete cytokines under physiological conditions or under stress. For instance, IL-6 was shown to be expressed by both beta and alpha cells in non-diabetic and autoantibody-positive individuals, but its expression was reduced in donors with T1D (24). Similarly, alpha cells were shown to express IL-1b in pancreatic tissue sections, irrespective of the diabetes status (25). Cytokines such as IL-6 act at a physiological level in islet cells to maintain glucose homeostasis (26), whereas other cytokines such as IFN-γ and CXCL10 may play a pathogenic role by contributing to immune cell recruitment and beta-cell killing. Cytokine receptors such as IL-4R, IL-13R, IL-6R were all shown to be widely expressed by islet cells (27–29), underscoring the possibility of cytokine-induced changes in islet cells that could facilitate immune attack. Here we will discuss some islet-intrinsic factors that may be involved in the recruitment of autoreactive T cells and secretion of pathogenic cytokines implicated in T1D.

Among the beta cell-intrinsic events, hyperexpression of MHC class I has been considered as a hallmark feature of T1D (30). Homing of autoreactive T cells to islets was inhibited in the absence of MHC class I expression in the NOD model of T1D (31). Treatment of human islets with IFN-γ induced MHC class I hyperexpression along with upregulation of chemokines such as CXCL10 in beta cells (32). Multiple reports identify pro-inflammatory cytokines and viral infection as major inducers of MHC class-I in islets. Although no viral infection has been shown to cause T1D directly, it may be possible that infection of beta cells may induce some islet–intrinsic changes. For instance, human islets upon infection with Coxsackie virus B (CVB) have been shown to secrete increased levels of IL-6, TNF, IP-10, and interferon-stimulated genes (33). Gallagher et al. have attempted to study CVB infection in an islet transplantation model devoid of native beta cells. Transplantation of healthy human islets in these mice reversed hyperglycemia, but this effect was abrogated upon infection of these mice with Coxsackievirus B. Signatures of viral RNA and increased levels of interferon-stimulated genes, CXCL10 and CCL5, could be observed in the transplanted islets (34). These evidence further prove that human islets could be infected with CVB, which could further drive pro-inflammatory factors and chemokines. The enrichment of autoreactive CD8 T cells near the islets suggests that they become attracted to their key antigen in insulin-containing islets during disease development (15), possibly due to the upregulation of MHC class I (30) and accumulation of target autoantigen (35). However, it is not clearly known whether MHC class I upregulation is responsible for the recruitment of autoreactive T cells or vice versa.

CXCL10 or IP-10 (IFN- inducible protein-10) is one of the most important cytokines implicated in T1D pathogenesis. CXCL10 expression could be observed in the beta cells of donors with recent-onset T1D, irrespective of their infection status with enterovirus. CXCR3, the receptor of CXCL10, could also be observed in proinsulin specific T cells of the T1D patient. In contrast, islets of non-diabetic donors were devoid of both CXCL10 and CXCR3 (36). Another independent study similarly reported that CXCL10 is expressed predominantly on beta cells and not by other endocrine cells in insulin-containing islets of T1D subjects (37). More recently, Nigi et al. have shown CXCL10 expression in alpha cells and beta cells of donors with T1D. Interestingly, insulin-containing islets in T1D had an increased percentage of CXCL10-positive alpha cells compared to CXCL-10-positive beta cells suggesting an involvement of alpha cells in chemokine secretion (38). Both CXCL9 and CXCL10 have been shown to be secreted by beta cells in insulitic islets, which drive CXCR3+ autoreactive T cells to islets in the RIP-LCMV model of T1D. The deletion of the CXCR3 gene in these mice has been beneficial in delaying the onset of T1D and insulitis (39). In addition, production of CXCL10 by autoreactive CD8 T cells was shown to be an essential factor in determining the diabetogenicity of CD8 T cell clones (40).

CCL21 has also been reported to be an essential factor for the homing of insulin-specific CD8 T cells to islets and for the interaction of islets with endothelial cells presenting the major auto-antigen, Insulin (31). Ectopic expression of CCL21 in beta cells influenced the recruitment of T cells, B cells, and dendritic cells to the islet periphery in a murine T1D model (41). Interestingly, expression of CCL21 by islets in NOD mice protected the mice from T1D; however, these mice still exhibited insulitis with T cells and fibroblastic reticular cell (FRC)–like cells expressing autoantigen, possibly inducing an antigen-specific immune tolerance (42).

In recent onset cases with T1D, laser capture microdissection studies have shown the overexpression of several interferon-stimulated genes such as *GBP1*, *TLR3*, *HLA-E*, and *STAT1* transcripts, compared to islets from non-diabetic individuals (43). Single-cell RNA sequencing of human islets revealed that chemokine transcripts were the most up-regulated among other transcripts upon exposure to pro-inflammatory cytokines. This report also confirmed the expression of cytokines and chemokines such as IL-6, IL-8, CCL2, and CXCL10 at the protein level (44). Exposure of human islets to cytokines, especially IL-1β and IFN-γ, has resulted in increased extracellular accumulation of proinsulin, suggesting its disproportionate conversion of proinsulin to insulin. This evidence points towards the role of cytokines in beta cell dysfunction (45). In contrast, the treatment of human islets with IFN-γ and IFN-α in-vitro has resulted in the upregulation of PDL1 by beta cells. PDL1 was also found to be expressed in insulin-containing islets from donors with T1D, but not in non-diabetic controls. Whether this phenomenon is a compensatory mechanism to reduce T cell activation is yet to be studied (46). Similarly, SOCS (suppressor of cytokine signaling) 1, 2, 3 were all expressed in the islets of donors with T1D, but not in non-diabetic donors. SOCS expression was up-regulated upon exposure of human islets to IFN-γ, TNFα, and IL-1β. Additionally, the transfection of SOCS1 in beta cells has been reported to inhibit IFN-γ signaling and MHC class I hyperexpression (47). This evidence suggests that beta cells could up-regulate SOCS in an attempt to protect the islets from cytokine-induced cell damage (48).

Immunohistochemical studies of donors with T1D revealed increased expression of some components of ER stress, such as CHOP and BIP, compared to non-diabetic individuals. These components mostly co-stained with insulin (49), implying that beta cells could be undergoing cellular stress during T1D. Human beta cells pre-treated with an ER stress inhibitor, TUDCA resulted in reduced cytokine-induced JNK pathway, IRE1α (inositol-requiring enzyme 1α) activity, a key component of unfolded protein response, and apoptosis (50).

Therefore, these events, in addition to other factors leading to islet-attraction of autoreactive CD8 T cells already within the pancreas, may be a crucial mechanism in T1D development.

## Concluding Questions

**Figure 1:**
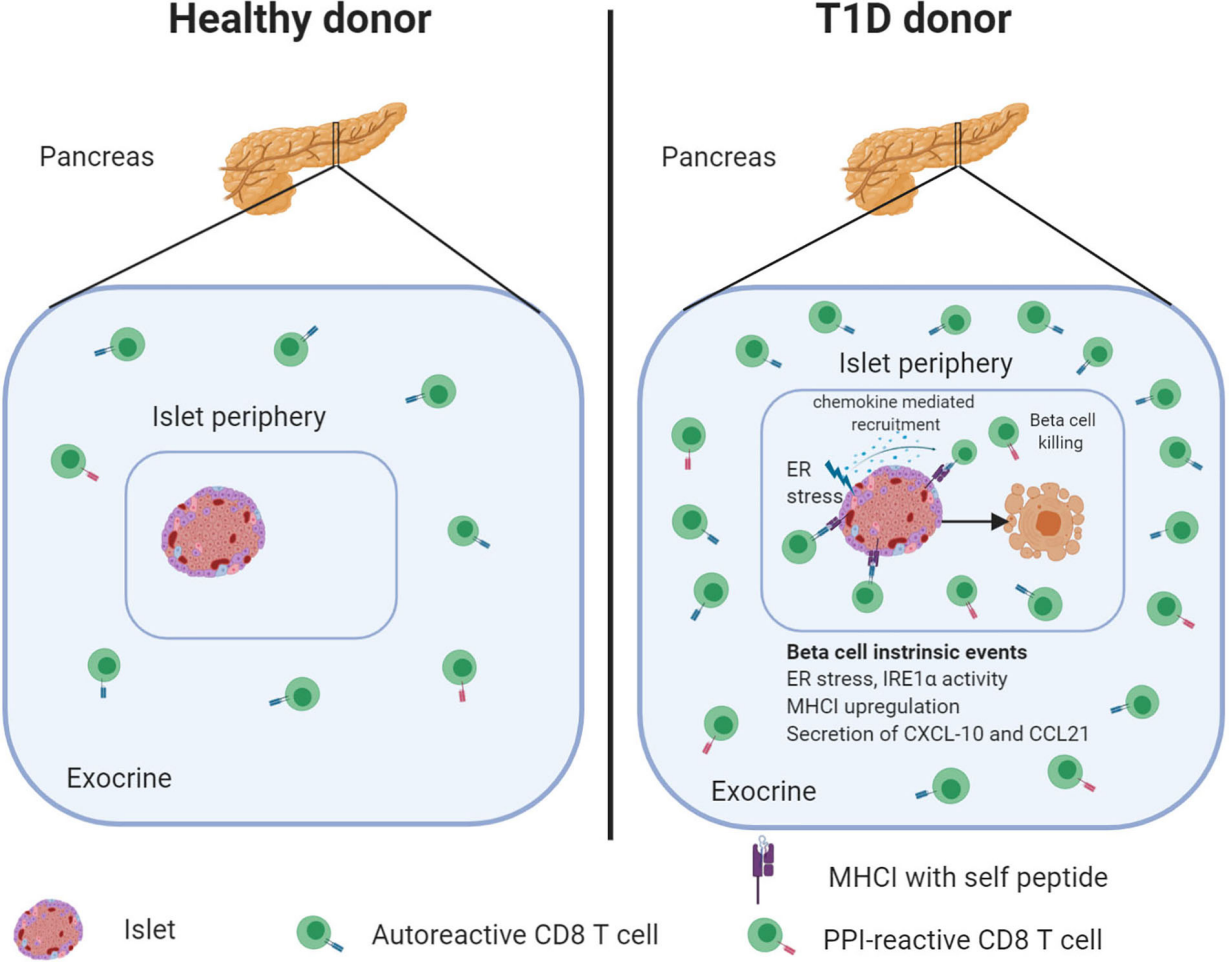
The healthy human pancreas contains autoreactive CD8 T cells that increase in numbers during diabetes development. Frequencies of PPI-specific CD8 T cells are similar in the exocrine pancreas irrespective of disease status. However, during disease progression, PPI-reactive CD8 T cells become attracted to the islets. The secretion of cytokines, under physiological conditions or stress, may contribute to immune cell recruitment and beta-cell killing. Created with BioRender.com.

In this review, we discussed the evidence that autoreactive CD8 T cells are an integral and large part of the pancreatic leukocyte population in healthy individuals and can be detected in large numbers in donors with T1D (**Figure 1**). Although autoreactive CD8 T cells are preferentially present in the pancreas, but not in blood of T1D patients, some key questions remain to be addressed.

1. Why are so many CD8 T cells in the exocrine pancreas PPI specific? Is it due to an abundance of this antigen in the pancreas? Could it be that beta-cells are not visible in healthy individuals under physiological conditions, a status termed immunological “ignorance” (51)? What then happens to these cells during disease development?
2. There is growing evidence from mouse and human studies suggesting insulin as the key autoantigen in T1D. But is PPI a “driver” autoantigen in T1D, and are PPI-specific T cells predominantly present in the pancreas of every individual, or does every individual have a different “driver” autoantigen?
3. Does CD8 T cell exhaustion occur in the pancreatic islets (indicated by PD-1 upregulation, and could this be a potential regulatory mechanism)? An exciting study recently demonstrated that islet-specific CD8 T cells of healthy controls and T1D patients comprise three dominant phenotypes that display characteristics of transitional memory and exhausted memory cells (52).
4. To date, less is known about post-translationally spliced peptides that can be recognized by CD8 T cells in the target organ. One study reported IAPP _15-17_/IAPP _5-10_ reactive cells in the human pancreas (9), but are there other neoantigens that can be recognized by CD8 T cells?
5. Is cytokine upregulation in beta cells necessarily a pathogenic factor in T1D or a compensatory mechanism to combat cellular stress?

To date, antigen-specific therapy for T1D that selectively dampens T cell responses to main beta-cell proteins remains elusive. One of the major hurdles is the choice of antigens and the timing of therapeutic intervention. Therefore, to design effective and durable immunotherapies for T1D, it is imperative to identify the numbers and phenotype of islet antigen-specific T cells, specifically in the pancreas, and whether changes in specificities occur over disease progression.

## Author Contributions

CB and SR wrote the manuscript. MV critically reviewed the manuscript. All authors contributed to the article and approved the submitted version.

## Funding

Funding was received from the National Institutes of Health (NIH), grant # RO1 AI092453 and RO1 AI134971.

## Conflict of Interest

MV is an employee of Novo Nordisk.

The remaining author declares that the research was conducted in the absence of any commercial or financial relationships that could be construed as a potential conflict of interest.
